# The high prevalence of myopia in Korean children with influence of parental refractive errors: The 2008-2012 Korean National Health and Nutrition Examination Survey

**DOI:** 10.1371/journal.pone.0207690

**Published:** 2018-11-26

**Authors:** Dong Hui Lim, Jisang Han, Tae-Young Chung, Sewoong Kang, Hyeon Woo Yim, on behalf of The Epidemiologic Survey Committee of the Korean Ophthalmologic Society

**Affiliations:** 1 Department of Ophthalmology, Samsung Medical Center, Sungkyunkwan University School of Medicine, Seoul, South Korea; 2 Graduate School, The Catholic University of Korea, Seoul, South Korea; 3 Department of Ophthalmology, Myongji Hospital, Seonam University School of Medicine, Goyang, South Korea; 4 Department of Preventive Medicine, College of Medicine, The Catholic University of Korea, Seoul, South Korea; National Yang-Ming University Hospital, TAIWAN

## Abstract

The present study was conducted to investigate the effect of parental refractive errors on myopic children in Korean families using a nationally representative survey. We used the ophthalmologic examination dataset of the Korean National Health and Nutrition Examination Surveys IV and V, a nationwide population-based cross-sectional study using a complex, stratified, multistage, probability cluster survey, which were performed from 2008–2012. We included 3,862 children from 5–18 years of age from 2,344 families without any ocular trauma, surgical history, or cataract affecting refractive errors. The generalized estimating equation was conducted to assess the association of refractive errors among children and their parents. Among 3,862 children, 2,495 had myopia, which was 64.6% prevalence. There were 208 children with high myopia (5.4%). The prevalence rate ratio (PRR) for pediatric myopia and high myopia with myopic parents was 1.34 (95% confidence intervals [CI] 1.24–1.45) and 3.11 (95% CI 1.93–5.01), respectively. The PRR of myopia and high myopia in children significantly increased to 1.37 (95% CI 1.04–1.81) and 11.41 (95% CI 6.24–20.88), as the degree of parental myopia increased (*P* < 0.001, respectively). Children with two myopic parents were more myopic than those with only one myopic parent (*P* < 0.001, respectively). In addition to parental myopia, the age of the child and household income were also significant risk factors for all degrees of pediatric myopia in a family (*P* ≤ 0.005, respectively). In conclusion, Korean children showed high prevalence of myopia. Children with myopic parents showed a significantly greater risk for myopia and high myopia.

## Introduction

Myopia is often considered benign, because it is easily corrected with glasses, contact lenses, or refractive surgery. However, the prevalence of myopia is rapidly increasing in East Asia, and the large social costs spent to correct myopia make the disorder a serious public health issue [1–12]. Furthermore, high myopia cannot be completely corrected and causes critical vision-threatening pathologies as well as blindness [1,13–17].

Multiple factors are involved in the development of myopia [18]. Both genetics and the environment play a role in the development and progression of myopia. Many epidemiological surveys have shown that excessive close-up work, a high level of education, and participation in fewer outdoor activities were important environmental risk factors for myopia [9,19–24]. Genetic factors have been proven to play a significant role in the development of myopia, and several genes are associated with high myopia [18,25–30]. There have been several studies to identify genetic contribution to myopia, and the risk of having myopic parents on pediatric myopia were reported greater at up to six times [31–38]. Previously, we found that myopia is highly heritable in a Korean population using a healthy twin study that included extended families as well as twins, which can exclude dominant genetic effects and consider all types of family relationship [25]. However, environment is considered to contribute more to the development of myopia compared to the genetics, and there is consensus that genes may determine susceptibility to environmental factors [39].

According to published reports, the prevalence of myopia did not exceed 50% in any of the regions in 2000, but, by 2050, the prevalence will be more than 50% in 57% of the countries, if current trends continue [39,40]. A shift to younger onset and rapid progression of myopia is accelerated worldwide, not just in East Asia. Generally early in onset and of high level, myopia has clearly familial proportion [41].

From this perspective, to evaluate the genetic influence on myopia and susceptibility to environment using a nationally representative data is meaningful. It is a great deal of interest whether children with myopic parents will or will not be myopic. In addition, familial clustering is a characteristic feature of myopia. Family members share genetic information and common environments, so determining how parental myopia acts on myopia in their offspring could help to assess the susceptibility for myopia. Thus, we evaluated the effect of parental refractive errors on pediatric myopia and high myopia, separately, and what part a shared environment explained in Korean families using the Korean National Health and Nutrition Examination Survey (KNHANES), a nationwide study.

## Methods

### Data source and study participants

**Fig 1:**
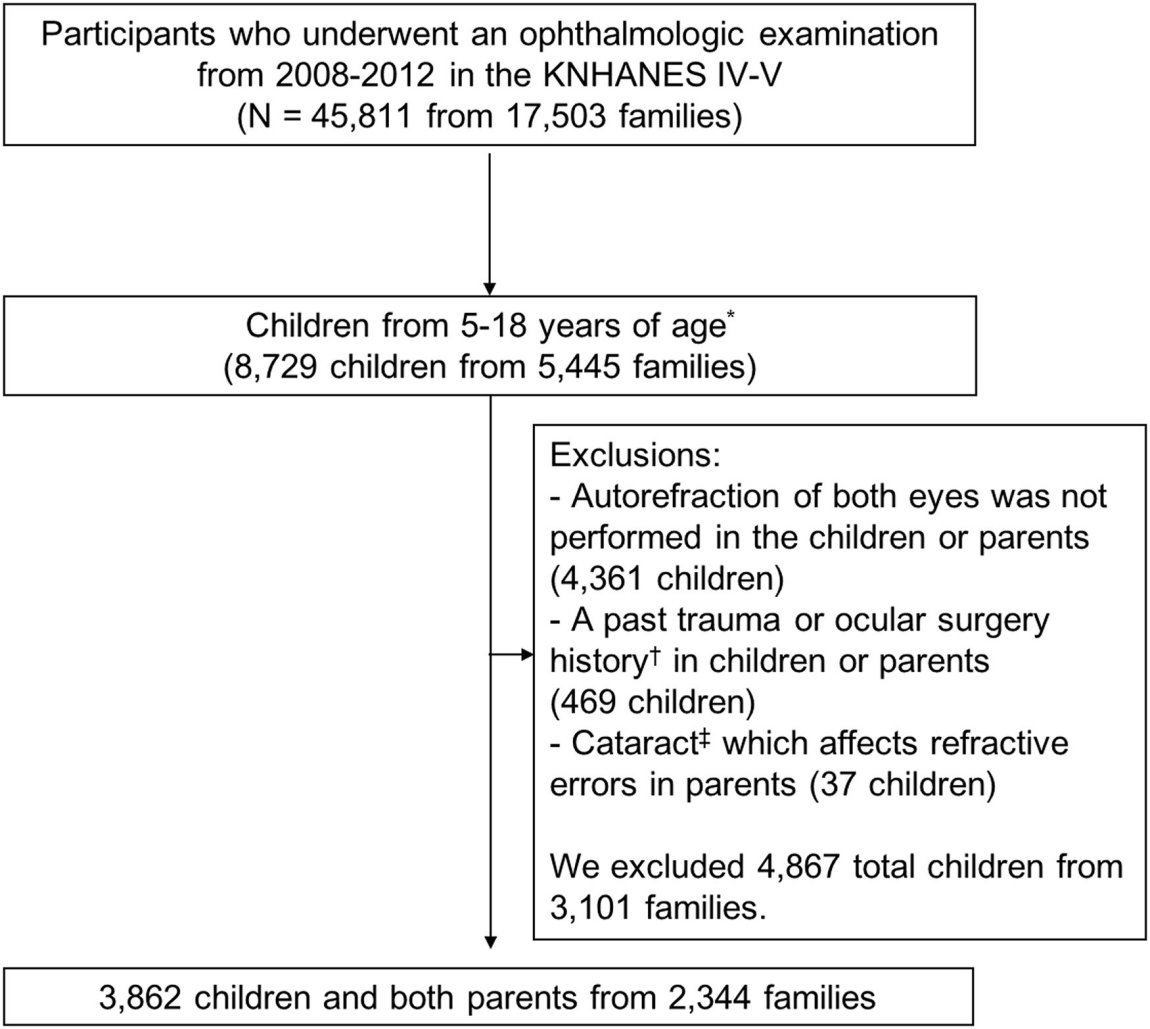
Identification of eligible participants from the 2008–2012 Korean National Health and Nutrition Examination Survey. KNHANES = Korean National Health and Nutrition Examination Survey; *The participants’ ages were not counted in Korean age, but in full. ^†^All types of surgery, including patients with pseudophakia, aphakia, or any history of refractive surgery. ^‡^Participants with cataract and a visual acuity of 20/25 or worse as assessed using a Snellen chart.

The KNHANES is a nationwide cross-sectional study that has been conducted by the Korea Centers for Disease Control and Prevention (KCDC) and the Korean Ministry of Health and Welfare since 1998. The survey uses a complex, stratified, multistage, probability cluster to obtain a representative sample of the population. The detailed study design of the KNHANES has been published previously [42], and the statistics of the KNHANES are publicly available on the study’s website (http://knhanes.cdc.go.kr. Accessed July 10. 2017). The KNHANES consists of three parts: a health interview, health examination, and nutrition survey. The health interview and examination are conducted by trained staff members in mobile centers that travel to each survey location around the country. The response rates of the health interview and examination were 74.3%, 79.2%, 77.5%, 76.1%, and 75.9% in 2008, 2009, 2010, 2011, and 2012, respectively. Ophthalmologic examinations were performed in all participants older than 3 years of age from July 2008 to 2012 in the fourth and fifth KNHANES [43]. Out of the total source population of 45,811 participants from 17,503 families, 8,729 children between 5 and 18 years of age from 5,445 families were eligible to participate in this study. Among children in two- or three-generation families that included both parents, 4,361 children who did not undergo autorefraction or without results of autorefraction in parents to evaluate refractive errors were excluded, as there were no additional available questionnaires for autorefraction results to estimate the parental and pediatric myopia status. 469 children who reported past trauma or surgical procedures, and 37 children who had parents with cataract that could influence refractive errors were excluded from the study. Finally, 3,862 children from 2,344 families consisting of a father, mother, and children were analyzed. The study population is described in detail in Fig 1. The Institutional Review Board of Samsung Medical Center approved the present study, which was conducted in accordance with the Declaration of Helsinki.

### Examinations and variable definitions

Ophthalmologic examinations were conducted by trained ophthalmologists. The quality of the ophthalmic survey was verified by the Epidemiologic Survey Committee of the Korean Ophthalmological Society. The ophthalmologists or ophthalmologic residents who participated in this survey were required to complete a training course and undergo supervised practice before working in the actual survey field [43]. The ophthalmic surgery or disease histories of the participants were assessed using survey questionnaires. Cataract was diagnosed with slit-lamp microscopic examinations and compared with Standardized Lens Opacities Classification System (LOCS) III photographic images. Examination with noncycloplegic automated refraction was carried out using an automated keratorefractometer (KR-8800, Topcon, Japan) with the fogging technique to reduce instrument myopia and patient accommodation. The mean of three consecutive measurements was recorded for each eye. The spherical equivalent (SE) refractive error was calculated as the sphere ± 1/2 cylinder. Mild myopia was defined as -3.0 < SE ≤ -0.5 diopters (D). Moderate myopia was set as -6.0 < SE ≤ -3.0 D, while high myopia was defined as SE ≤ -6.0 D [44,45].

Body mass index (BMI) was calculated as body weight (kg) / height (m)^2^. Parental education level was classified into two categories in mother and father, respectively; equal to or less than high school and equal to or higher than undergraduate. Household income was categorized into tertiles from lowest to highest. The participants’ areas of residence were classified as either urban or rural. The question regarding sunlight exposure (more than five hours or less a day) was asked only of participants who were at least 19 years old, and we evaluated vitamin D level as an objective indicator of outdoor activities. In the KNHANES, vitamin D level was measured only in participants 10 years of age or older, and then the variable was analyzed separately.

### Statistical analysis

The SE values in both eyes were highly correlated in Pearson’s correlation analysis (*r* = 0.9272, *P* <0.0001), and the potential risk factors were assessed by subject, not by eye. Therefore, in this study, we used the right eye for the analysis.

The KNHANES sample weight was used to adjust for oversampling, nonresponse, and the overall Korean population from 2008–2012, and the data were analyzed using SAS software version 9.4 for Windows (SAS Institute Inc., Cary, NC, USA). Continuous variables were reported as median and range values, while categorical variables were reported as number and frequency. Chi-square tests and the Wilcoxon signed rank sum test with Dwass, Steel, Critchlow-Fligner post hoc analysis were used for statistical comparison of continuous and categorical variables, respectively. We imputed the missing values for BMI using the iterative Bayesian MCMC algorithm in SAS PROC MI with the maximum likelihood EM algorithm used as the prior estimates for the MCMC algorithm. Because siblings with the same father and mother would violate the assumption of independence when using the proc surveylogistic procedure to analyze the correlation of refractive errors between parents and children, families were clustered. To assess the effect of parental myopia on their child’s myopia while accounting for correlations among family members, we constructed separate generalized estimating equation (GEE) models for the spherical equivalent of each parent-children pair. GEEs for binary outcome with link log functions, Poisson distribution, and link cumulative logit functions with a multinomial distribution for ordinal outcome were performed. Age, sex, vitamin D level, child’s BMI, parents’ highest education level, household income, and area of residence were analyzed as possible risk factors for pediatric myopia using a univariable GEE model. Factors with a *P* value < 0.2 in a univariable GEE analysis were simultaneously adjusted in an analysis using a multivariable GEE model. The standard errors of estimates were calculated, and *P-*values less than 0.05 were considered statistically significant.

## Results

### General characteristics of the participants

This study included 3,862 children from 2,344 families. The mean age was 43.0 ± 5.5 (range: 27–65) years for fathers, 40.2 ± 5.3 (range: 22–57) years for mothers, and 11.1 ± 3.8 (range: 5–18) years for children. The children’s school levels were distributed as follows: 547 preschool age, 1,845 elementary school students, 830 middle school students, and 640 high school students. There were 2,033 (52.6%) sons and 1,829 (47.4%) daughters. The mean SEs of fathers, mothers, and children were -1.43 ± 1.95 (-11.63 to +3.31) D, -1.60 ± 2.09 (-23.56 to +2.56) D, and -1.74 ± 2.20 (-15.43 to +5.63) D, respectively. Age, BMI, child’s vitamin D level, parental refractive errors, mother’s highest education level, and household income showed statistically significant differences among the mild, moderate and high myopic children groups (*P* = 0.006 for vitamin D level of child, *P* = 0.007 for mother’s highest education level, *P* = 0.007 for household income, and *P*<0.001 for age, BMI of the child, and parental refractive errors). The characteristics of participants by refractive errors of the children are summarized in Table 1.

**Table 1:** Characteristics of the study participants according to children’s refractive errors.

	Children’s refractive errors				*P-*value
	SE* > -0.5 D None (n = 1,367)	-3.0 < SE ≤ -0.5 D Mild (n = 1,553)	-6.0 < SE ≤ -3.0 D Moderate (n = 734)	SE ≤ -6.0 D High myopia (n = 208)	
*Children’s parameters (n = 3*,*862)*					
Gender					
Male (n = 2,033)	736(53.8)	827(53.3)	370(50.4)	100(48.1)	0.238
Female (n = 1,829)	631(46.2)	726(46.8)	364(49.6)	108(51.9)	
Age (years)					
Mean(SD)	8.9(3.5)	11.6(3.5)	13.3(3.0)	14.6(2.6)	<0.001
Median(range)	8.0(5.0–18.0)	11.0(5.0–18.0)	13.0(5.0–18.0)	15.0(5.0–18.0)	
BMI ‡(kg/m^2^)					
Mean(SD)	17.8(3.2)	19.2(3.6)	20.3(3.7)	20.9(3.7)	<0.001
Median(Range)	16.9(12.5–39.1)	18.6(12.6–34.7)	19.8(12.7–35.7)	20.3(13.7–32.6)	
Vitamin D (ng/ml) (n = 2,191)†					
Mean(SD)	17.8(5.9)	17.3(5.7)	17.0(5.1)	16.2(5.2)	0.006
Median(range)	16.9(6.3–43.5)	16.5(5.7–37.7)	16.6(5.8–35.3)	15.4(7.5–37.1)	
*Parental parameters (n = 2*,*344)*	*n = 731*	*n = 932*	*n = 523*	*n = 158*	
Refractive errors (D)					
Father					
Mean(SD)	-1.2(1.7)	-1.4(2.0)	-1.7(2.1)	-2.0(2.2)	<0.001
Median(range)	-0.7(-10.1–3.1)	-0.6(-11.3–3.3)	-0.9(-11.6–2.6)	-1.3(-8.7–1.4)	
SE > -0.5 D (n = 945)	317(43.4)	394(42.3)	184(35.2)	50(31.7)	<0.001
-3.0 < SE ≤ -0.5 D (n = 974)	312(42.7)	378(40.6)	225(43.0)	59(37.3)	
-6.0 < SE ≤ -3.0 D (n = 336)	84(11.5)	125(13.4)	90(17.2)	37(23.4)	
SE ≤ -6.0 D (n = 89)	18(2.5)	35(3.8)	24(4.6)	12(7.6)	
Mother					
Mean(SD)	-1.4(1.9)	-1.5(1.9)	-1.8(2.2)	-2.5(3.1)	<0.001
Median(range)	-0.8(-15.4–2.6)	-0.9(-11.9–2.6)	-0.9(-11.1–1.7)	-1.2(-23.6–1.0)	
SE > -0.5 D (n = 801)	274(37.5)	323(34.7)	166(31.7)	38(24.1)	<0.001
-3.0 < SE ≤ -0.5 D (n = 1,091)	328(44.9)	458(49.1)	236(45.1)	69(43.7)	
-6.0 < SE ≤ -3.0 D (n = 344)	109(14.9)	117(12.6)	86(16.4)	32(20.3)	
SE ≤ -6.0 D (n = 108)	20(2.7)	34(3.7)	35(6.7)	19(12.0)	
Highest education level					
Father					
≤High school (n = 1,159)	358(49.0)	477(51.2)	254(48.6)	70(44.3)	0.381
≥Undergraduate (n = 1,146)	358(49.0)	443(47.5)	262(50.1)	83(52.5)	
Unknown (n = 39)	15(2.1)	12(1.3)	7(1.3)	5(3.2)	
Mother					
≤High school (n = 1,429)	406(55.5)	598(64.2)	317(60.6)	108(68.4)	0.007
≥Undergraduate (n = 902)	320(43.8)	331(35.5)	202(38.6)	49(31.0)	
Unknown (n = 13)	5(0.7)	3(0.3)	4(0.8)	1(0.6)	
Household income					
Lower (n = 113)	39(5.3)	50(5.4)	20(3.8)	4(2.5)	0.007
Middle (n = 1,422)	469(64.2)	561(60.2)	297(56.8)	95(60.1)	
Higher (n = 793)	215(29.4)	318(34.1)	204(39.0)	56(35.4)	
Unknown (n = 16)	8(1.1)	3(0.3)	2(0.4)	3(1.9)	
Area of residence					
Urban (n = 1,974)	608(83.2)	797(85.5)	436(83.4)	133(84.2)	0.559
Rural (n = 370)	123(16.8)	135(14.5)	87(16.6)	25(15.8)	

SE = spherical equivalent; D = diopters; SD = standard deviations; BMI = body mass index

Mild myopia: -3.0 < SE ≤ -0.5 D ; moderate myopia: -6.0 < SE ≤ -3.0 D ; high myopia: SE ≤ -6.0 D

* Spherical equivalents were calculated as the spherical value + (cylindrical value/2).

^†^ Vitamin D level was measured only in participants 10 years of age or older.

^‡^ Missing BMI data from 289 participants were imputed into SAS using the MCMC method of Proc MI.

Values are n (%) for categorical variables or mean (standard deviations), median (range) for continuous variables. *P-*values were calculated using the Chi-square test, Fisher’s exact test or Wilcoxon rank sum test.

### Prevalence of myopia and high myopia according to parental myopia in Korean children

Among 3,862 children, 2,495 had myopia (64.6% prevalence). The prevalence of pediatric myopia that was mild or more severe increased from 57.4% when the SEs of both parents were >-0.5 D to 68.2% when both parents were ≤-0.5 D, from 61.6% when the SEs of both parents were >-3.0 D to 75.5% when both parents were ≤-3.0 D, and from 63.4% when the SEs of both parents were >-6.0 D to 87.5% when both parents were ≤ -6.0 D. The differences were statistically significant for all values (*P*<0.001).

In pediatric high myopia, effects of parental myopia on children were observed more obviously. The prevalence of pediatric high myopia increased from 3.3% when the SEs of both parents were >-0.5 D to 7.3% when both parents were ≤-0.5 D, and from 4.1% when the SEs of both parents were >-3.0 D to 17.9% when both parents were ≤-3.0 D. The prevalence of pediatric high myopia increased from 4.8% when neither parent was highly myopic to 50.0% if the mother and father had high myopia (*P*<0.001). The prevalence rates of myopia and high myopia in children according to parental myopia are shown in S1 Table.

### Association of parental and pediatric myopia in Korean families

Children with myopic parents were more likely to be myopic than those without myopic parents. The prevalence rate ratios (PRR) for pediatric myopia and high myopia with myopic parents was 1.34 (95% confidence intervals [CI] 1.24 to 1.45) and 3.11 (95% CI 1.93 to 5.01), respectively. The PRR of myopia and high myopia in children significantly increased to 1.37 (95% CI 1.04 to 1.81) and 11.41 (95% CI 6.24 to 20.88), respectively, as the degree of parental myopia increased (*P* < 0.001, respectively).

In detail, the crude PRR of pediatric myopia with SE value ≤-0.5 D comparing one parent with myopia and both parents with myopia to no parental myopia were 1.11 (95% CI 1.02 to 1.21), and 1.17 (95% CI 1.08 to 1.28), respectively (*P*<0.001). After adjusting for the age of the child, the PRR (95% CI) values were 1.18 (1.09–1.27), and 1.35 (1.25–1.46), respectively (*P*<0.001). Age strengthened the association between parental and pediatric myopia. In a multivariable model after simultaneously adjusting for age, sex, BMI of the child, mother’s highest education level, and household income with a *P-*value < 0.2 in a univariable analysis (S2 Table), the corresponding estimates were 1.17 (1.08–1.26), and 1.34 (1.24–1.45), respectively (*P*<0.001). These findings were consistent after adjustment for potential confounders but slightly weakened the association between parental and pediatric myopia, suggesting the influence of common environmental effects on pediatric myopia within a family. As parental myopia increased from mild (SE ≤ -0.5 D) to moderate (SE ≤ -3.0 D) and severe (SE ≤ -6.0 D), the crude and adjusted PRRs also increased, and a significant association between pediatric and parental myopia was also shown (Table 2).

**Table 2:** Prevalence rate ratios and 95% confidence intervals for pediatric myopia (SE ≤ -0.5 D) according to the presence of parental myopia.

	Univariable			Multivariable					
	Crude PRR (95% CI)	*p-*value	*p-*value for trend	Adjusted* PRR (95% CI)	*p-*value	*p-*value for trend	Adjusted† PRR (95% CI)	*p-*value	*p-*value for trend
*Parental myopia (SE ≤ -0*.*5 D)*									
No (n = 554)	Reference			Reference			Reference		
One parent (n = 1,740)	1.11(1.02–1.21)	0.021	<0.001	1.18(1.09–1.27)	<0.001	<0.001	1.17(1.08–1.26)	<0.001	<0.001
Both parents (n = 1,568)	1.17(1.08–1.28)	<0.001		1.35(1.25–1.46)	<0.001		1.34(1.24–1.45)	<0.001	
*Parental myopia(SE ≤ -3*.*0 D)*									
No (n = 2,612)	Reference			Reference			Reference		
One parent (n = 1,066)	1.13(1.07–1.20)	<0.001	<0.001	1.24(1.18–1.30)	<0.001	<0.001	1.23(1.17–1.29)	<0.001	<0.001
Both parents (n = 184)	1.22(1.10–1.34)	<0.001		1.33(1.21–1.45)	<0.001		1.32(1.20–1.45)	<0.001	
*Parental myopia(SE ≤ -6*.*0 D)*									
No (n = 3,550)	Reference			Reference			Reference		
One parent (n = 304)	1.23(1.15–1.32)	<0.001	<0.001	1.28(1.20–1.37)	<0.001	<0.001	1.27(1.19–1.36)	<0.001	<0.001
Both parents (n = 8)	1.36(1.00–1.84)	0.051		1.41(1.08–1.85)	0.013		1.37(1.04–1.81)	0.027	

PRR = prevalence rate ratio; CI = confidence interval; SE = spherical equivalent; D = diopters

Mild myopia: -3.0 < SE ≤ -0.5 D ; moderate myopia: -6.0 < SE ≤ -3.0 D ; high myopia: SE ≤ -6.0 D

Spherical equivalents were calculated as the spherical value + (cylindrical value/2).

*adjusted for the age of the children

^†^adjusted multivariable analysis if the *p-*value was <0.2 in the univariable analysis (age, sex, and BMI of the children, mother’s highest education level, and household income).

PRR estimates were calculated using estimating equations (GEE) with link log functions and the Poisson distribution, which included family clustering variables.

Testing for trends was performed using a continuous version of the parental myopia status entered in the same models.

The trend was similar in highly myopic children with ≤-6.0 D of SE (Table 3); age strengthened the parental effect on pediatric high myopia, while other environmental factors slightly weakened the association. In high myopia, age, sex, and BMI of the child, mother’s highest education level, and household income were also potential confounders with a *P-*value < 0.2 in a univariable analysis for pediatric high myopia. Having only one parent with mild myopia was not found to be a significant factor for high myopia in children (*P* = 0.127).

**Table 3:** Prevalence rate ratios and 95% confidence intervals for pediatric high myopia (SE ≤ -6.0 D) according to the presence of parental myopia.

	Univariable			Multivariable					
	Crude PRR (95% CI)	*p-*value	*p-*value for trend	Adjusted* PRR (95% CI)	*p-*value	*p-*value for trend	Adjusted† PRR (95% CI)	*p-*value	*p-*value for trend
*Parental myopia (SE ≤ -0*.*5 D)*									
No (n = 554)	Reference			Reference			Reference		
One parent (n = 1,740)	1.31(0.79–2.16)	0.302	<0.001	1.54(0.95–2.51)	0.081	<0.001	1.46(0.90–2.38)	0.127	<0.001
Both parents (n = 1,568)	2.19(1.34–3.58)	0.002		3.27(2.04–5.23)	<0.001		3.11(1.93–5.01)	<0.001	
*Parental myopia(SE ≤ -3*.*0 D)*									
No (n = 2,612)	Reference			Reference			Reference		
One parent (n = 1,066)	1.59(1.16–2.18)	0.004	<0.001	2.09(1.54–2.82)	<0.001	<0.001	2.09(1.54–2.85)	<0.001	<0.001
Both parents (n = 184)	4.36(2.96–6.41)	<0.001		5.73(4.07–8.07)	<0.001		5.90(4.14–8.40)	<0.001	
*Parental myopia(SE ≤ -6*.*0 D)*									
No (n = 3,550)	Reference			Reference			Reference		
One parent (n = 304)	2.25(1.51–3.34)	<0.001	<0.001	2.63(1.83–3.79)	<0.001	<0.001	2.60(1.80–3.76)	<0.001	<0.001
Both parents (n = 8)	10.07(5.19–19.53)	<0.001		12.22(6.90–21.66)	<0.001		11.41(6.24–20.88)	<0.001	

PRR = prevalence rate ratio; CI = confidence interval; SE = spherical equivalent; D = diopters

Mild myopia: -3.0 < SE ≤ -0.5 D ; moderate myopia: -6.0 < SE ≤ -3.0 D ; high myopia: SE ≤ -6.0 D

Spherical equivalents were calculated as the spherical value + (cylindrical value/2).

*adjusted for the age of the children

^†^adjusted multivariable analysis if the *p-*value was <0.2 in the univariable analysis (age, sex, and BMI of the children, mother’s highest education level, and household income).

PRR estimates were calculated using estimating equations (GEE) with link log functions and the Poisson distribution, which included family clustering variables.

Testing for trends was performed using a continuous version of the parental myopia status entered in the same models.

### Common environmental risk factors for pediatric myopia in a Korean family

In a univariable analysis, age, sex, BMI of the child, mother’s highest education level, and household income were found to be possible common environmental factors that influence the development of pediatric myopia in addition to parental myopia in a family. In a fully adjusted multivariable model, the statistically significant risk factors for children’s refractive errors when comparing mild, moderate and high myopia groups to a non-myopic group were the child’s age, household income, and all degrees of parental myopia (*P*≤0.005; Table 4). Child’s age and household income showed reliably significant effects on pediatric myopia after adjusting for myopia in one or both parent in three different categories: mild (SE ≤ -0.5 D), moderate (SE ≤ -3.0 D) and severe (SE ≤ -6.0 D), while the sex and BMI of the child and the mother’s highest education level occasionally demonstrated significant influences on children’s refractive errors according to the degrees of parental myopia. In a subgroup analysis with 2,191 children 10 years of age or older who had data regarding vitamin D level, the age and BMI of the child, household income and parental myopia showed constantly significant effects on pediatric myopia in a fully adjusted multivariable model for children’s refractive errors when comparing mild, moderate and high myopia groups to a non-myopic group (S3 Table).

**Table 4:** Risk factors for pediatric myopia categorized as none = SE > -0.5 D, mild = -3.0 < SE ≤ -0.5 D, moderate = -6.0 < SE ≤ -3.0 D, and high myopia = SE ≤ -6.0 D, according to the different levels of parental myopia, which is mild (SE ≤ -0.5 D), moderate (SE ≤ -3.0 D) and severe (SE ≤ -6.0 D).

	Crude PRR (95% CI)	*p-*value	Adjusted† PRR (95% CI)	*p-*value	Adjusted† PRR (95% CI)	*p-*value	Adjusted† PRR (95% CI)	*p-*value
Age of children	1.31(1.28–1.33)	<0.001	1.33(1.30–1.36)	<0.001	1.32(1.29–1.35)	<0.001	1.31(1.28–1.34)	<0.001
Sex of children								
Male (n = 2,033)	Reference							
Female (n = 1,829)	1.11(0.99–1.25)	0.076	1.15(1.01–1.30)	0.031	1.12(0.99–1.27)	0.075	1.13(1.00–1.28)	0.052
BMI of children	1.16(1.14–1.18)	<0.001	1.02(1.00–1.04)	0.062	1.03(1.00–1.05)	0.023	1.03(1.00–1.05)	0.017
Father’s highest education level								
≤High school (n = 1,159)	Reference							
≥Undergraduate (n = 1,146)	1.07(0.94–1.22)	0.326						
Unknown (n = 39)	0.91(0.54–1.55)	0.739						
Mother’s highest education level								
≤High school (n = 1,429)	Reference							
≥Undergraduate (n = 902)	0.85(0.74–0.97)	0.018	1.13(0.97–1.30)	0.112	1.05(0.91–1.21)	0.522	1.18(1.02–1.36)	0.024
Unknown (n = 13)	0.92(0.37–2.26)	0.854	1.30(0.56–3.03)	0.542	1.57(0.63–3.92)	0.331	1.38(0.56–3.40)	0.481
Household income								
Lower (n = 113)	Reference		Reference		Reference		Reference	
Middle (n = 1,422)	1.26(0.94–1.71)	0.126	1.62(1.18–2.22)	0.003	1.71(1.26–2.33)	0.001	1.73(1.28–2.34)	<0.001
Higher (n = 793)	1.65(1.21–2.25)	0.002	1.60(1.15–2.22)	0.005	1.74(1.27–2.40)	0.001	1.79(1.31–2.45)	<0.001
Unknown (n = 16)	1.00(0.41–2.41)	0.992	0.83(0.30–2.27)	0.710	1.07(0.42–2.77)	0.886	1.13(0.41–3.08)	0.814
Area of residence								
Urban (n = 1,974)	Reference							
Rural (n = 370)	0.90(0.76–1.07)	0.247						
*Parental myopia (SE ≤ -0*.*5 D)*								
No (n = 554)	Reference		Reference					
One parent (n = 1,740)	1.37(1.13–1.67)	0.001	1.72(1.39–2.13)	<0.001				
Both parents (n = 1,568)	1.76(1.44–2.14)	<0.001	2.96(2.37–3.69)	<0.001				
*Parental myopia(SE ≤ -3*.*0 D)*								
No (n = 2,612)	Reference				Reference			
One parent (n = 1,066)	1.49(1.29–1.73)	<0.001			2.11(1.81–2.46)	<0.001		
Both parents (n = 184)	3.18(2.22–4.56)	<0.001			5.12(3.60–7.30)	<0.001		
*Parental myopia(SE ≤ -6*.*0 D)*								
No (n = 3,550)	Reference						Reference	
One parent (n = 304)	2.34(1.80–3.03)	<0.001					2.99(2.31–3.86)	<0.001
Both parents (n = 8)	7.96(1.78–35.59)	0.007					10.74(2.68–43.04)	0.001

PRR = prevalence rate ratio; CI = confidence interval; SE = spherical equivalent; D = diopters

The outcome variable (pediatric myopia) was categorized as follows: none = SE > -0.5 D, mild = -3.0 < SE ≤ -0.5 D, moderate = -6.0 < SE ≤ -3.0 D, and high myopia = SE ≤ -6.0 D

^†^adjusted multivariable analysis if the *p*-value was <0.2 in the univariable analysis (age, sex and BMI of the children, mother’s highest education level, and household income). Different regression models adjusting for different levels of parental myopia.

PRR estimates were calculated using estimating equations (GEE) with link cumulative logit functions and a multinomial distribution that included family clustering variables.

Testing for trends was performed using a continuous version of the parental myopia status entered in the same models.

## Discussions

We comprehensively investigated the effect of parental refractive errors on pediatric myopia, including high myopia. In Korean children from 5–18 years of age, the prevalence of myopia (SE ≤ -0.5 D) was 64.6%, and that of high myopia (SE ≤ -6.0 D) was 5.4%. All degrees of parental myopia were strongly associated with the prevalence of myopic children in Korea. Children with two myopic parents were more likely to be myopic compared to children with one myopic parent, who, in turn, were more likely to be myopic than children with no myopic parents (*P*<0.001). The children with myopic parents showed statistically significant high PRR for myopia and high myopia, which was 1.34 (95% CI 1.24 to 1.45) and 3.11 (95% CI 1.93 to 5.01), respectively. As the level of parental myopia increased, the PRR of children’s myopia and high myopia increased accordingly to 1.37 (95% CI 1.04 to 1.81) and 11.41 (95% CI 6.24 to 20.88), respectively. The PRRs for pediatric myopia and high myopia with myopic parents were all statistically significant (*P*<0.001).

Several studies have shown that parental myopia has a significant effect on the development and progression of pediatric myopia [33–37]. In particular, the effects of having two myopic parents were significantly higher than those for children with one or no myopic parents [26,35]. One study showed that children with two myopic parents were 6.42 times more likely to become myopic as children with one or no myopic parents [38].

The genetic contribution to the development of myopia is considered relatively small compared to the environment recently, although it is a great deal of interest whether children with myopic parents will or will not be myopic. However, there is consensus that genes may determine susceptibility to environmental factors [39]. As myopia is rapidly increasing worldwide, to assess the susceptibility for myopia is important. Early detection of risk factors for myopic development and intervention to slow its progression are essential to prevent permanent and complicated myopic changes. In this sense, the results of the present study using the KNHANES, a large nationwide representative sample of Koreans, could help to suggest a guide for young children with all degrees of myopic parents as they age.

Furthermore, we evaluated many common environmental factors shared by family members as well as genetic factors of parental refractive errors. Age, sex, BMI of the child, mother’s highest education level, and household income were revealed to have important effects on the prevalence of pediatric myopia and high myopia. The age of child and household income also had constantly significant effects on child’s myopia after a full adjustment for possible confounding factors (*P* ≤ 0.005), including myopia in one or both parents in three different categories (mild (SE ≤ -0.5 D), moderate (SE ≤ -3.0 D) and severe (SE ≤ -6.0 D)), while the sex and BMI of the child, and the mother’s highest education level occasionally showed significant influences on child’s refractive errors according to the degrees of parental myopia.

We could not assess the direct relationship between the amount of close-up work, outside activities, and sun exposure, which are known risk factors for development and progression of myopia, using the KNHANES because the data were not available or were limited in the children [46]. As a surrogate variable for sun exposure, we evaluated the association of vitamin D level with myopia in children [22,47]. There was a significant relationship between vitamin D level and myopia. However, vitamin D was measured only in participants 10 years of age or older, so this variable was analyzed separately. In a subgroup analysis of children with data regarding vitamin D level, this variable was revealed to have a significant effect on pediatric myopia; however, the effect was weakened after adjusting for other statistically significant risk factors including age and BMI of the child, maternal and paternal highest education level, household income, and area of residence. We can therefore assume that myopia can be weakened or strengthened by both genetic and shared common environmental effects among family members, although myopia should be related with a large amount of indoor study time as previously reported [47].

Age is known to be one of the most important risk factors related to the effect of axial growth of the eyeball. The age of myopia onset is generally from 5–15 years, and myopia is the most common eye disease among children [48]. Children older than 11 years old showed a significantly high prevalence of myopia. According to previous studies, the prevalence of myopia among young adults in Singapore ranges from 85–90% [7,8]. In Korea, several epidemiologic studies have recently reported the prevalence of myopia to be 53.7% across all ages and 78.8% among children from 12–18 years [23,49]. High myopia ≤-6.0 D, which can lead to severe pathologic eye conditions and vision loss, including retinal detachment, myopic macular degeneration, cataract, or glaucoma, had a 21.62% prevalence in 19-year-old Korean males [13–17,23]. Generally early in onset and of high level, myopia has clearly familial proportion [41].

In the current study, child’s risk for myopia significantly increased as the education level of the mother increased in the presence of high myopia in one or both parents. We can therefore postulate that more educated parents are more myopic and had children at a later age, and the children are more myopic. BMI also showed a close relationship with myopia in children in a multivariable analysis after adjustment children’s age, suggesting that obese children who take part in fewer outside activities or perform tasks that involve increased close-up work are more at risk for myopic refractive errors. Since time outdoors was not included in the multivariate models, it is possible that time outdoors may have confounded the association between BMI and myopia.

As the KNHANES is a cross-sectional study, the strength of an association between parental and pediatric myopia was estimated using a PRR. The use of the PRR as an estimate of the incidence ratio is subject to bias, especially when the disease of interest does not have a low prevalence. Myopia is common among Korean children, so the possibility that a PRR tends to underestimate the strength of the association between the exposure and the outcome should be considered when interpreting these results.

Another possible bias might have been caused by the non-biological relationship between some parents and children. In the KNHANES, there was no questionnaire to assess whether the respondents were biological offspring or stepchildren. However, the proportion of stepfamilies in Korea is much lower than that in Western countries. According to the Korean Child-Youth Well-Being Index (KCWI) report (available at http://www.korsofa.org; accessed April 2, 2017), 0.7% of school children lived in stepfamilies, which was lower than 9.25%, the average of Organization for Economic Co-organization and Development countries according to Health Behavior in School-aged Children (HBSC) report (available at http://www.hbsc.org; accessed April 2, 2017). Therefore, any bias caused by a non-biological relationship between parents and children in stepfamilies might not be significant in the present study.

In the study, 3,862 children from 2,344 families were included in the current study, they represent only 44% of the total 8,729 children from 5,445 families that make up the eligible population of the KNHANES. To confirm the representativeness of our results without bias, we compared the demographics between the study population and the excluded population. Between these two groups, there were no statistical differences for all variables, except for household income. The study population included a larger proportion of higher level of household income families. The household income has a statistical relationship with the parental myopia and causal relationship with the pediatric myopia, therefore, the possibility of confounding effects for household income should be considered.

Socioeconomic status (SES) was assessed by household income. As household income is higher, pediatric myopia increased. The results should be interpreted carefully considering the possibility of overestimation. As described above, the study population included a larger proportion of higher level of household income families.

There are several limitations of the present study. First, the KNHANES is a cross-sectional study, so its results cannot guarantee a causal relationship. Second, parental and child’s refractive error was determined via noncycloplegic refraction in KNHANES as with US NHANES. Unfortunately, the KNHANES was not designed specifically for an ophthalmological evaluation of the children. So, the refractive error may be over-minus which may have resulted in an over-estimated of the myopia prevalence. However, the association of parental and pediatric myopia was robust and consistent throughout all analyzes. Third, we were unable to examine the direct effects of well-known environmental factors, including close-up work, outside activities, or sun exposure, because the KNHANES did not assess these items in children. Instead, we adjusted the environmental factors which reflect outdoor activities and near work as much as possible including BMI, parental educational level, household income, areas of residence, and vitamin D. Further studies are required with full consideration for myopia-associated environmental risk factors. Stepchildren also could not be identified in the KNHANES questionnaires, and the biological relationship between parents and children was not guaranteed. Fourth, our study included a larger proportion of higher level of household income families among the KNHANES population, so the association could be overestimated because of the confounding effect of household income. Lastly, ethnic and regional differences should be considered to generalize our results to other populations.

Nevertheless, we identified large numbers of family clustering risk factors for myopia, such as maternal educational levels or household incomes, which could be used to adjust for potential confounders in a multivariable analysis. In addition, the large and well-controlled sample from the KNHANES provided sufficient power to detect an association between parental and pediatric myopia and the effects of child’s age and household income on myopia in Korean children. We tried not to introduce bias and to preserve the representativeness of the population methodologically.

In conclusion, we found a strong association between parental and pediatric myopia as well as an effect of older child age and household income on myopic children in Korean families. Further studies should be conducted to confirm our findings and reveal any causal relationships.

## Supporting information

S1 Table Prevalence of myopia (SE ≤ -0.5 D) or high myopia (SE ≤ -6.0 D) in Korean children according to parental myopia. (DOCX)

S2 Table Univariable analysis of potential risk factors for pediatric myopia (SE ≤ -0.5 D) and high myopia (SE ≤ -6.0 D). (DOCX)

S3 Table Risk factors for pediatric myopia categorized as none = SE > -0.5 D, mild = -3.0 < SE ≤ -0.5 D, moderate = -6.0 < SE ≤ -3.0 D, and high myopia = SE ≤ -6.0 D, according to the different levels of parental myopia, which is mild (SE ≤ -0.5 D), moderate (SE ≤ -3.0 D) and severe (SE ≤ -6.0 D): A subgroup analysis with 2,191 children of 10 years of age or older who measured vitamin D level. (DOCX)
