# Correction: The high prevalence of myopia in Korean children with influence of parental refractive errors: The 2008-2012 Korean National Health and Nutrition Examination Survey

**DOI:** 10.1371/journal.pone.0209876

**Published:** 2018-12-20

**Authors:** Dong Hui Lim, Jisang Han, Tae-Young Chung, Sewoong Kang, Hyeon Woo Yim, on behalf of The Epidemiologic Survey Committee of the Korean Ophthalmologic Society

The second affiliation for the second author is not indicated. Jisang Han is also affiliated with: Department of Ophthalmology, Kangbuk Samsung Hospital, Sungkyunkwan University School of Medicine, Seoul, South Korea.
