# Supplementary material for: The high prevalence of myopia in Korean children with influence of parental refractive errors: The 2008-2012 Korean National Health and Nutrition Examination Survey
Source: PLoS One. 2018 Nov 26;13(11):e0207690. doi: 10.1371/journal.pone.0207690 (PMC6261017; doi:10.1371/journal.pone.0207690)
Supplement: S1 Table — (DOCX) [file pone.0207690.s001.docx]

**S1 Table. Prevalence of myopia (SE ≤ -0.5 D) or high myopia (SE ≤ -6.0 D) in Korean children according to parental myopia.**

|  | Pediatric myopia (mild + moderate + high; SE ≤ -0.5 D) | | | | Pediatric high myopia (SE ≤ -6.0 D) | | | |
| --- | --- | --- | --- | --- | --- | --- | --- | --- |
|  | Non- myopia (n=1,367) | ≥ Mild myopia (n=2,495) | *p-*value | *p*-value for trend | Non-high myopia (n=3,654) | High myopia (n=208) | *p-*value | *p-*value for trend |
| *Parental myopia (SE ≤ -0.5 D)* |  |  |  |  |  |  |  |  |
| No mild myopia (n=554) | 236(42.6) | 318(57.4) | <0.001 | <0.001 | 536(96.8) | 18(3.3) | <0.001 | <0.001 |
| Paternal mild myopia (n=748) | 279(37.3) | 469(62.7) |  |  | 719(96.1) | 29(3.9) |  |  |
| Maternal mild myopia (n=992) | 353(35.6) | 639(64.4) |  |  | 946(95.4) | 46(4.6) |  |  |
| Mild myopia in both parents  (n=1,568) | 499(31.8) | 1,069(68.2) |  |  | 1,453(92.7) | 115(7.3) |  |  |
| *Parental myopia(SE ≤ -3.0 D)* |  |  |  |  |  |  |  |  |
| No moderate myopia (n=2,612) | 1,003(38.4) | 1,609(61.6) | <0.001 | <0.001 | 2,506(95.9) | 106(4.1) | <0.001 | <0.001 |
| Paternal moderate myopia (n=504) | 143(28.4) | 361(71.6) |  |  | 474(94.1) | 30(6.0) |  |  |
| Maternal moderate myopia (n=562) | 176(31.3) | 386(68.7) |  |  | 523(93.1) | 39(6.9) |  |  |
| Moderate myopia in both parents  (n=184) | 45(24.5) | 139(75.5) |  |  | 151(82.1) | 33(17.9) |  |  |
| *Parental myopia(SE ≤ -6.0 D)* |  |  |  |  |  |  |  |  |
| No high myopia (n=3,550) | 1,300(36.6) | 2,250(63.4) | <0.001 | <0.001 | 3,379(95.2) | 171(4.8) | <0.001 | <0.001 |
| Paternal high myopia (n=134) | 32(23.9) | 102(76.1) |  |  | 124(92.5) | 10(7.5) |  |  |
| Maternal high myopia (n=170) | 34(20.0) | 136(80.0) |  |  | 147(86.5) | 23(13.5) |  |  |
| High myopia in both parents (n=8) | 1(12.5) | 7(87.5) |  |  | 4(50.0) | 4(50.0) |  |  |

SE = spherical equivalent; D = diopters

Mild myopia : -3.0 < SE ≤ -0.5 D ; moderate myopia : -6.0 < SE ≤ -3.0 D ; high myopia : SE ≤ -6.0 D

Spherical equivalents were calculated as the spherical value + (cylindrical value/2).
Data are presented as n (%).
*P-*values were calculated using the Chi-square test or Fisher's exact test, Cochran-Armitage test for trend.
