# Supplementary material for: The high prevalence of myopia in Korean children with influence of parental refractive errors: The 2008-2012 Korean National Health and Nutrition Examination Survey
Source: PLoS One. 2018 Nov 26;13(11):e0207690. doi: 10.1371/journal.pone.0207690 (PMC6261017; doi:10.1371/journal.pone.0207690)
Supplement: S2 Table — (DOCX) [file pone.0207690.s002.docx]

**S2 Table. Univariable analysis of potential risk factors for pediatric myopia (SE ≤ -0.5 D) and high myopia (SE ≤ -6.0 D)**

|  | Pediatric myopia (mild + moderate + high; SE ≤ -0.5 D) | | | | Pediatric high myopia (SE ≤ -6.0 D) | | | |
| --- | --- | --- | --- | --- | --- | --- | --- | --- |
|  | Non- myopia (n=1,367) | ≥ Mild myopia (n=2,495) | Crude PRR  (95% CI) | *p-*value | Non-high myopia (n=3,654) | High myopia (n=208) | Crude PRR  (95% CI) | *p-*value |
| Age of children | 8.9(3.5) | 12.4(3.4) | 1.09(1.08-1.09) | <0.001 | 10.9(3.8) | 14.6(2.6) | 1.30(1.25-1.35) | <0.001 |
| Sex of children |  |  |  |  |  |  |  |  |
| Male | 736(36.2) | 1,297(63.8) | Reference |  | 1,933(95.1) | 100(4.9) | Reference |  |
| Female | 631(34.5) | 1,198(65.5) | 1.03(0.99-1.08) | 0.170 | 1,721(94.1) | 108(5.9) | 1.20(0.92-1.56) | 0.170 |
| BMI of children | 17.8(3.2) | 19.6(3.7) | 1.05(1.04-1.05) | <0.001 | 18.9(3.6) | 20.9(3.7) | 1.12(1.09-1.15) | <0.001 |
| Father’s highest education level |  |  |  |  |  |  |  |  |
| ≤High school | 673(35.8) | 1,209(64.2) | Reference |  | 1,791(95.2) | 91(4.8) | Reference |  |
| ≥Undergraduate | 671(34.9) | 1,254(65.1) | 1.01(0.96-1.06) | 0.783 | 1,813(94.2) | 112(5.8) | 1.18(0.89-1.57) | 0.242 |
| Unknown | 23(41.8) | 32(58.2) | 0.90(0.72-1.12) | 0.350 | 50(90.9) | 5(9.1) | 1.88(0.80-4.38) | 0.146 |
| Mother’s highest education level |  |  |  |  |  |  |  |  |
| ≤High school | 794(33.6) | 1,566(66.4) | Reference |  | 2,222(94.2) | 138(5.9) | Reference |  |
| ≥Undergraduate | 566(38.2) | 918(61.9) | 0.92(0.87-0.97) | 0.004 | 1,415(95.4) | 69(4.7) | 0.79(0.58-1.07) | 0.128 |
| Unknown | 7(38.9) | 11(61.1) | 0.91(0.60-1.37) | 0.648 | 17(94.4) | 1(5.6) | 1.03(0.15-7.09) | 0.978 |
| Household income |  |  |  |  |  |  |  |  |
| Lower | 76(40.6) | 111(59.4) | Reference |  | 183(97.9) | 4(2.1) | Reference |  |
| Middle | 372(40.1) | 555(59.9) | 1.04(0.91-1.19) | 0.541 | 874(94.3) | 53(5.7) | 2.27(0.84-6.09) | 0.105 |
| Higher | 511(35.4) | 931(64.6) | 1.15(1.01-1.32) | 0.035 | 1,370(95.0) | 72(5.0) | 2.58(0.95-7.01) | 0.064 |
| Unknown | 396(30.9) | 885(69.1) | 0.86(0.58-1.28) | 0.455 | 1,205(94.1) | 76(5.9) | 5.73(1.32-24.88) | 0.020 |
| Area of residence |  |  |  |  |  |  |  |  |
| Rural | 1,139(35.1) | 2,104(64.9) | Reference |  | 3,066(94.5) | 177(5.5) | Reference |  |
| Urban | 228(36.8) | 391(63.2) | 0.97(0.91-1.05) | 0.476 | 588(95.0) | 31(5.0) | 0.96(0.66-1.39) | 0.814 |

SE = spherical equivalent; D= diopters; PRR = prevalence rate ratio; CI = confidence interval; BMI = body mass index

Mild myopia : -3.0 < SE ≤ -0.5 D ; moderate myopia : -6.0 < SE ≤ -3.0 D ; high myopia : SE ≤ -6.0 D

Spherical equivalents were calculated as the spherical value + (cylindrical value/2).

Values are n (%) for categorical variables or mean (standard deviations) for continuous variables.
PRR estimates were calculated using estimating equations (GEE) with link log functions and the Poisson distribution, which included family clustering variables.
