# Supplementary material for: The high prevalence of myopia in Korean children with influence of parental refractive errors: The 2008-2012 Korean National Health and Nutrition Examination Survey
Source: PLoS One. 2018 Nov 26;13(11):e0207690. doi: 10.1371/journal.pone.0207690 (PMC6261017; doi:10.1371/journal.pone.0207690)
Supplement: S3 Table — (DOCX) [file pone.0207690.s003.docx]

**S3 Table. Risk factors for pediatric myopia categorized as none = SE > -0.5 D, mild = -3.0 < SE ≤ -0.5 D, moderate = -6.0 < SE ≤ -3.0 D, and high myopia = SE ≤ -6.0 D, according to the different levels of parental myopia, which is mild (SE ≤ -0.5 D), moderate (SE ≤ -3.0 D) and severe (SE ≤ -6.0 D): A subgroup analysis with 2,191 children of 10 years of age or older who measured vitamin D level.**

|  | Crude PRR  (95% CI) | *p-*value | Adjusted PRR†  (95% CI) | *p-*value | Adjusted PRR†  (95% CI) | *p-*value | Adjusted PRR†  (95% CI) | *p-*value |
| --- | --- | --- | --- | --- | --- | --- | --- | --- |
| Age of children | 1.17(1.13-1.21) | <0.001 | 1.19(1.14-1.23) | <0.001 | 1.19(1.15-1.24) | <0.001 | 1.18(1.14-1.22) | <0.001 |
| Sex of children |  |  |  |  |  |  |  |  |
| Male | Reference |  |  |  |  |  |  |  |
| Female | 1.10(0.94-1.28) | 0.250 |  |  |  |  |  |  |
| BMI of children | 1.06(1.04-1.09) | <0.001 | 1.03(1.00-1.05) | 0.022 | 1.03(1.01-1.06) | 0.011 | 1.04(1.01-1.06) | 0.005 |
| Father’s highest education level |  |  |  |  |  |  |  |  |
| ≤High school | Reference |  | Reference |  | Reference |  | Reference |  |
| ≥undergraduate | 1.25(1.05-1.48) | 0.013 | 1.11 (0.90-1.36) | 0.344 | 1.10(0.90-1.35) | 0.339 | 1.17(0.95-1.43) | 0.136 |
| Unknown | 1.10(0.49-2.48) | 0.814 | 0.93 (0.39-2.19) | 0.866 | 1.01(0.42-2.40) | 0.986 | 0.95(0.40-2.28) | 0.914 |
| Mother’s highest education level |  |  |  |  |  |  |  |  |
| ≤High school | Reference |  | Reference |  | Reference |  | Reference |  |
| ≥Undergraduate | 1.17(0.97-1.40) | 0.101 | 1.10(0.89-1.37) | 0.389 | 1.03(0.83-1.29) | 0.766 | 1.16(0.93-1.44) | 0.190 |
| Unknown | 2.99(0.89-10.11) | 0.077 | 2.67(0.68-10.55) | 0.161 | 3.67(1.04-12.93) | 0.043 | 2.99(0.80-11.21) | 0.105 |
| Household income |  |  |  |  |  |  |  |  |
| Lower | Reference |  | Reference |  | Reference |  | Reference |  |
| Middle | 1.59(1.11-2.29) | 0.012 | 1.62(1.11-2.38) | 0.013 | 1.72(1.19-2.50) | 0.004 | 1.69(1.18-2.41) | 0.004 |
| Higher | 1.80(1.24-2.61) | 0.002 | 1.51(1.02-2.24) | 0.041 | 1.60(1.09-2.36) | 0.017 | 1.66(1.14-2.39) | 0.008 |
| Unknown | 0.83(0.22-3.10) | 0.787 | 0.57(0.17-1.95) | 0.373 | 0.74(0.21-2.64) | 0.648 | 0.74(0.22-2.56) | 0.640 |
| Area of residence |  |  |  |  |  |  |  |  |
| Rural | Reference |  | Reference |  | Reference |  | Reference |  |
| Urban | 0.85(0.68-1.06) | 0.144 | 0.97(0.77-1.22) | 0.791 | 0.94(0.75-1.18) | 0.587 | 0.94(0.75-1.18) | 0.588 |
| Vitamin D (ng/ml) | 0.98(0.96-0.99) | 0.001 | 1.00(0.98-1.02) | 0.983 | 1.00(0.98-1.01) | 0.832 | 1.00(0.98-1.01) | 0.854 |
| *Parental myopia (SE ≤ -0.5 D)* |  |  |  |  |  |  |  |  |
| No | Reference |  | Reference |  |  |  |  |  |
| One parent | 1.62(1.28-2.06) | <0.001 | 1.64(1.28-2.10) | <0.001 |  |  |  |  |
| Both parents | 2.59(2.02-3.33) | <0.001 | 2.78(2.13-3.63) | <0.001 |  |  |  |  |
| *Parental myopia(SE ≤ -3.0 D)* |  |  |  |  |  |  |  |  |
| No | Reference |  |  |  | Reference |  |  |  |
| One parent | 2.01(1.65-2.45) | <0.001 |  |  | 2.22(1.18-2.72) | <0.001 |  |  |
| Both parents | 6.21(3.86-9.98) | <0.001 |  |  | 7.27(4.49-11.77) | <0.001 |  |  |
| *Parental myopia(SE ≤ -6.0 D)* |  |  |  |  |  |  |  |  |
| No | Reference |  |  |  |  |  | Reference |  |
| One parent | 2.90(2.12-3.97) | <0.001 |  |  |  |  | 3.27(2.36-4.51) | <0.001 |
| Both parents | infinity |  |  |  |  |  | infinity |  |

PRR = prevalence rate ratio; CI = confidence interval; BMI = body mass index; SE = spherical equivalent; D= diopters

The outcome variable (pediatric myopia) was categorized as follows: none = SE > -0.5 D, mild = -3.0 < SE ≤ -0.5 D, moderate = -6.0 < SE ≤ -3.0 D, and high myopia = SE ≤ -6.0 D
†adjusted multivariable analysis if the *p*-value was <0.2 in the univariable analysis (age, BMI and vitamin D level of the children, maternal and paternal highest education level, household income, and area of residence). Different regression models adjusting for different levels of parental myopia.
PRR estimates were calculated using estimating equations (GEE) with link cumulative logit functions and a multinomial distribution that included family clustering variables.
Testing for trends was performed using a continuous version of the parental myopia status entered in the same models.
